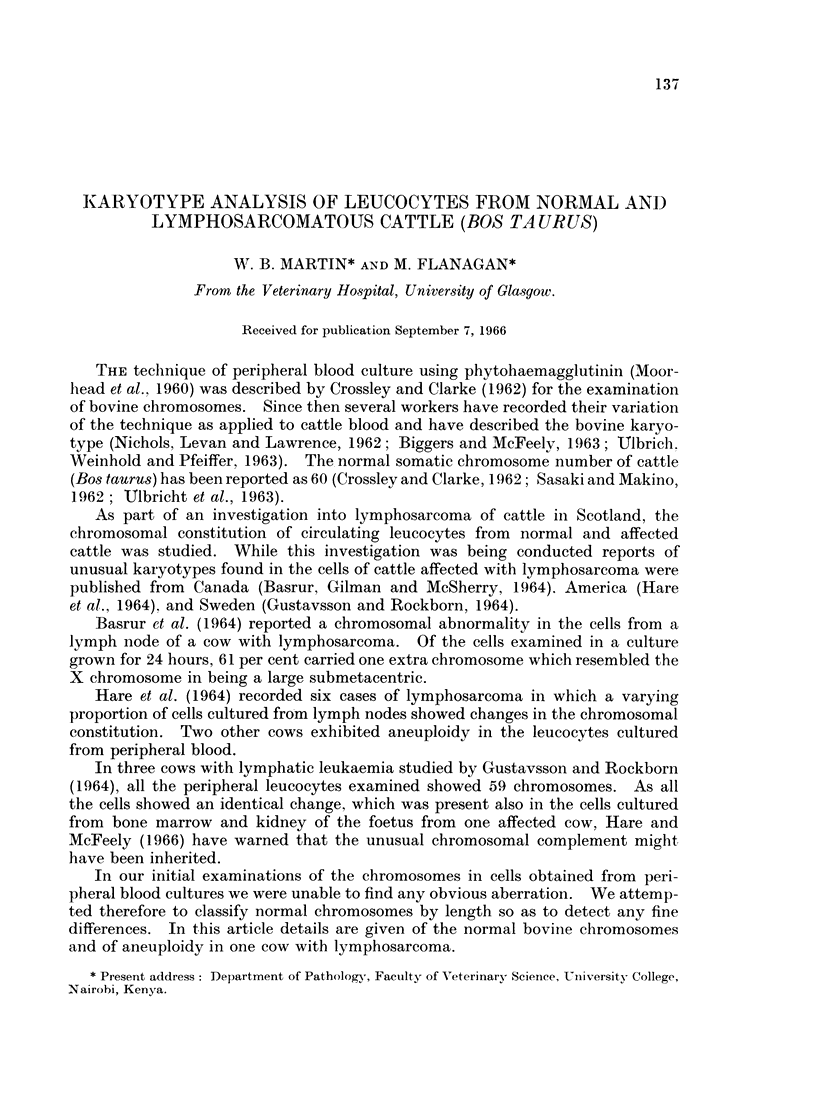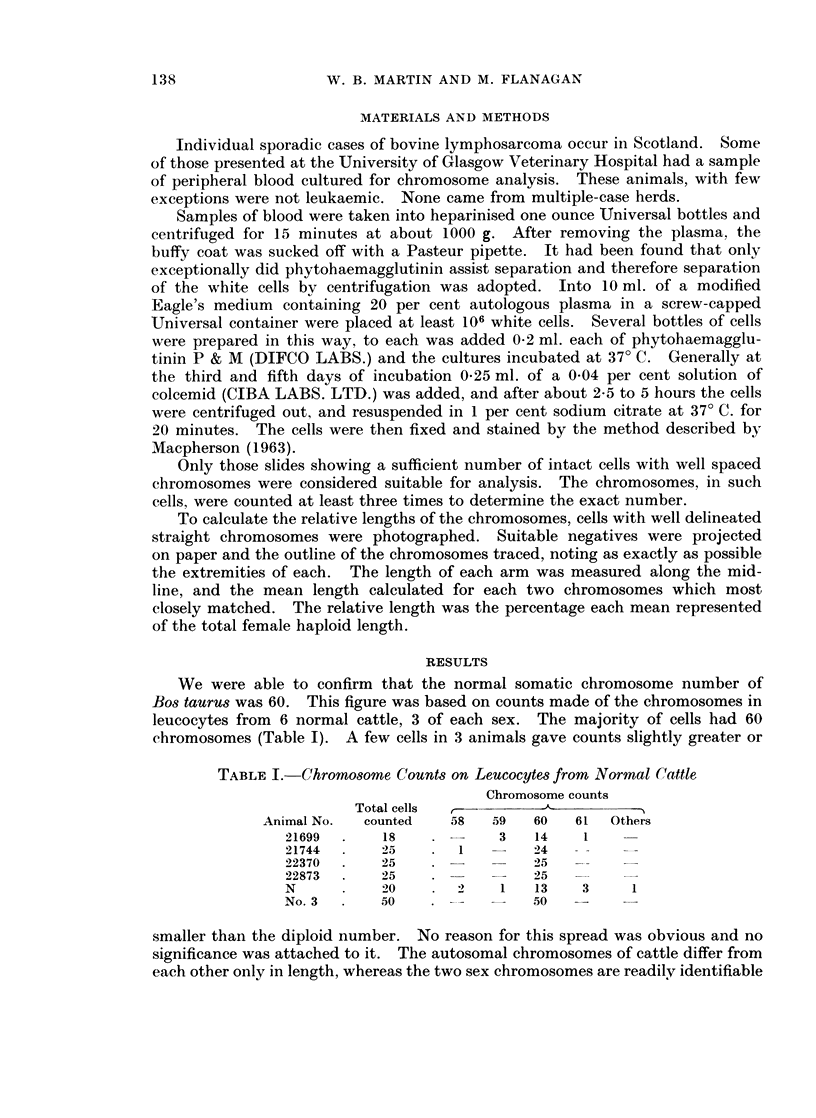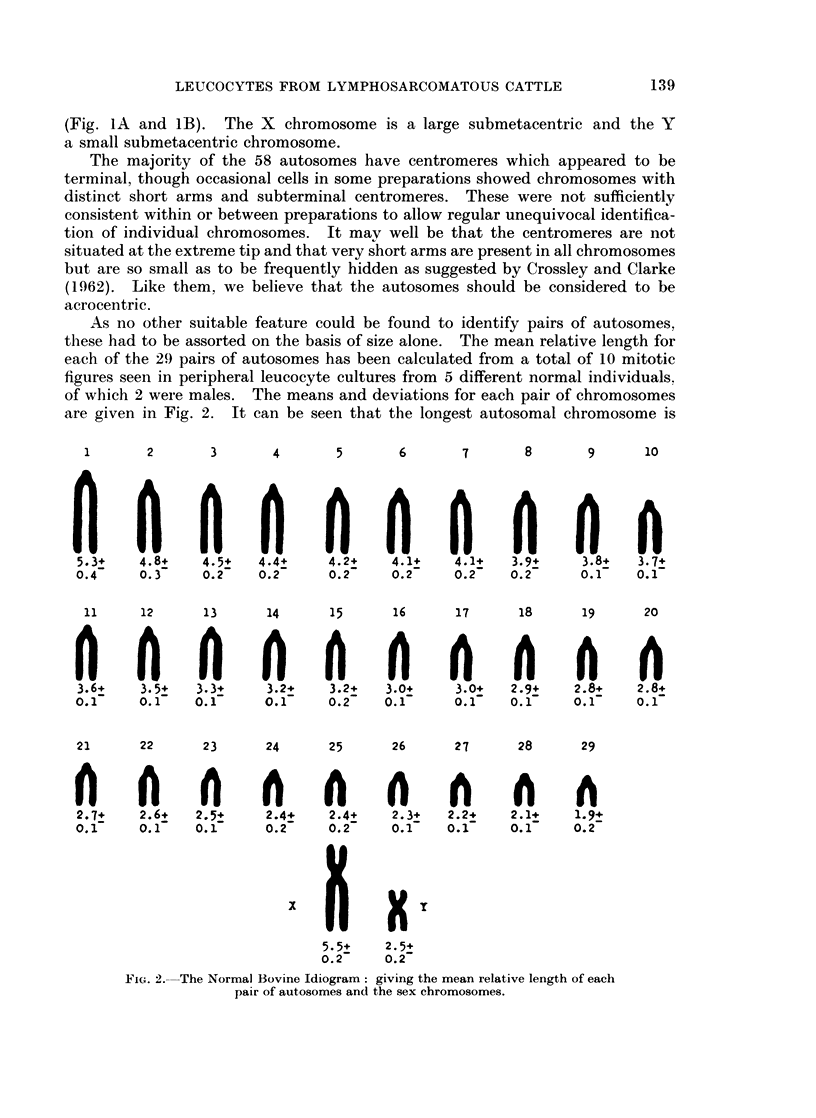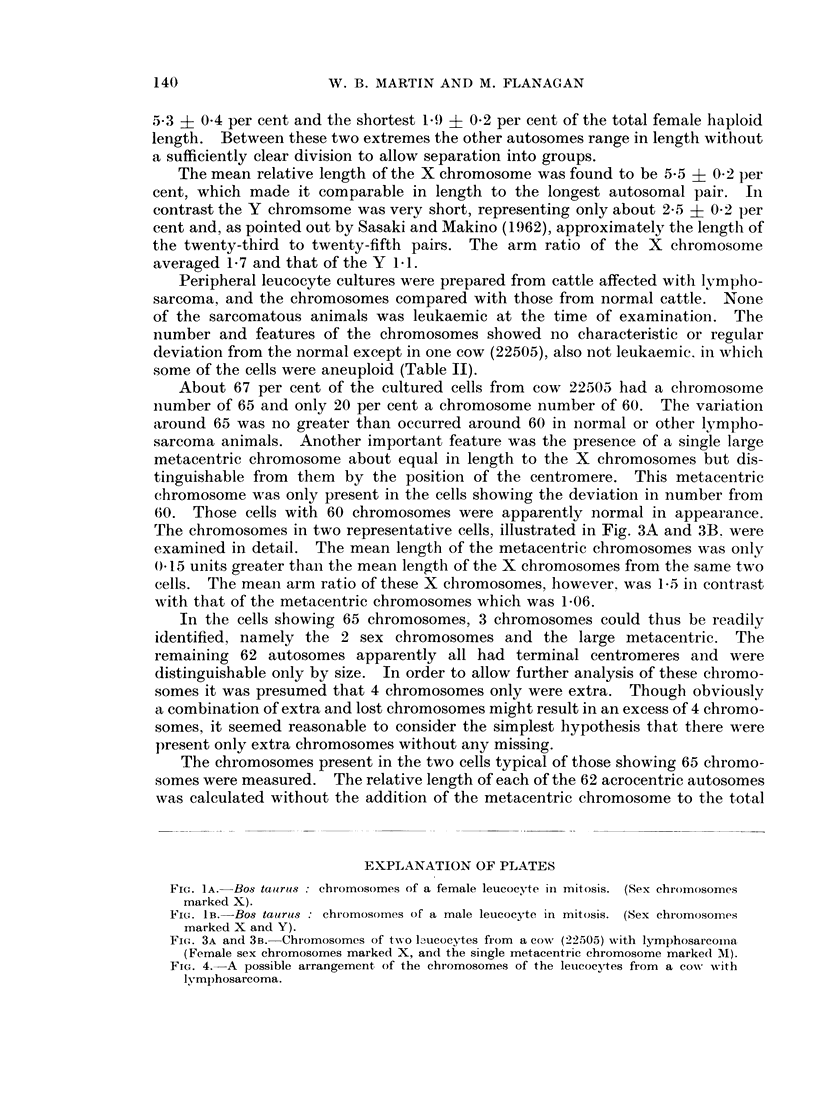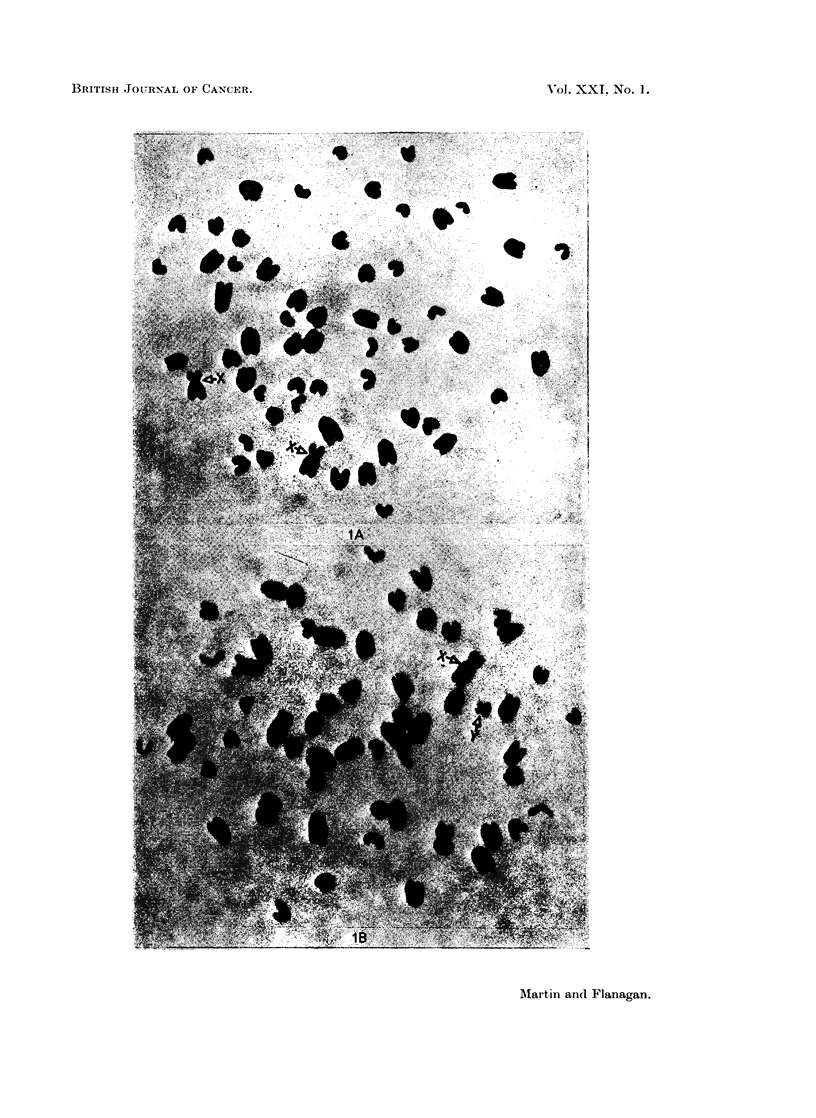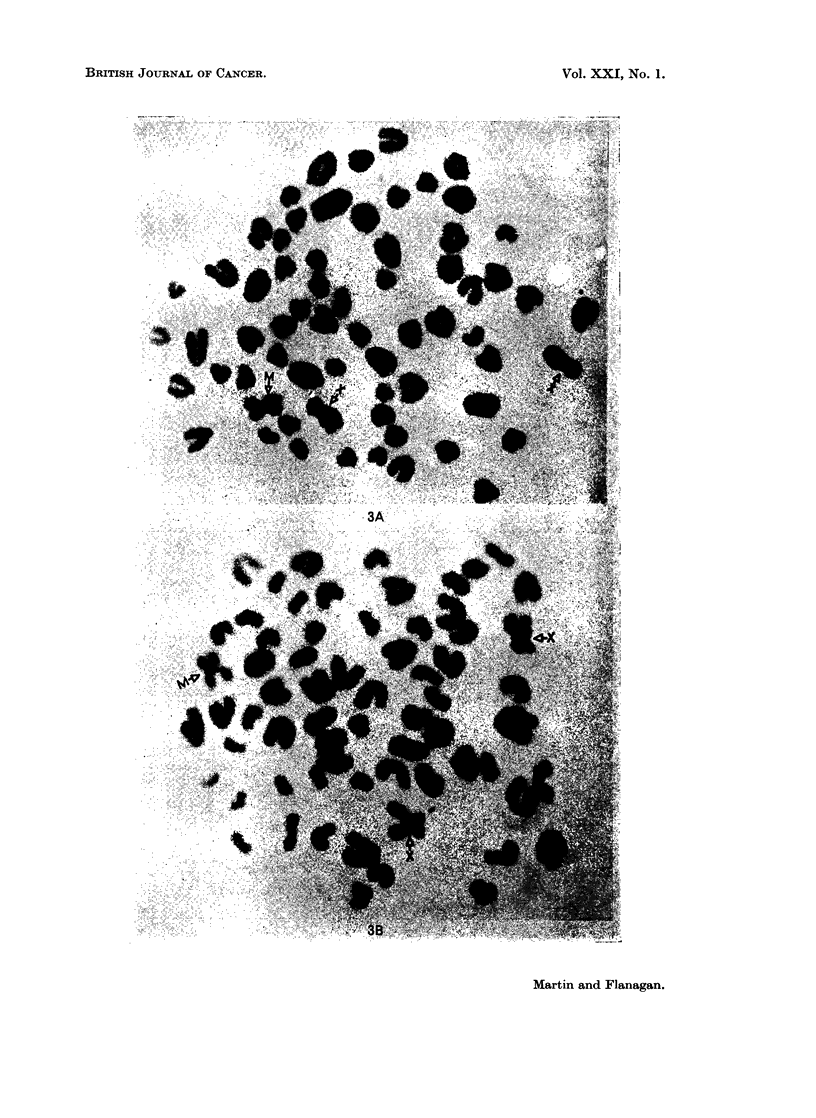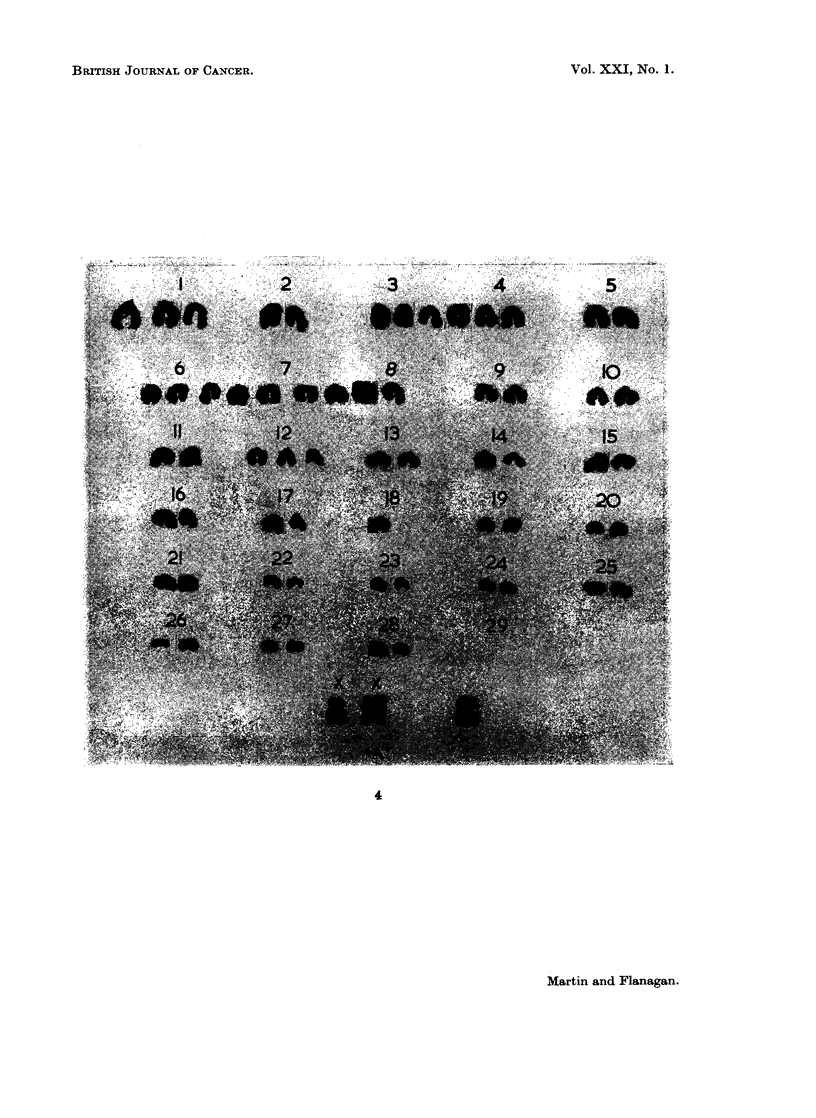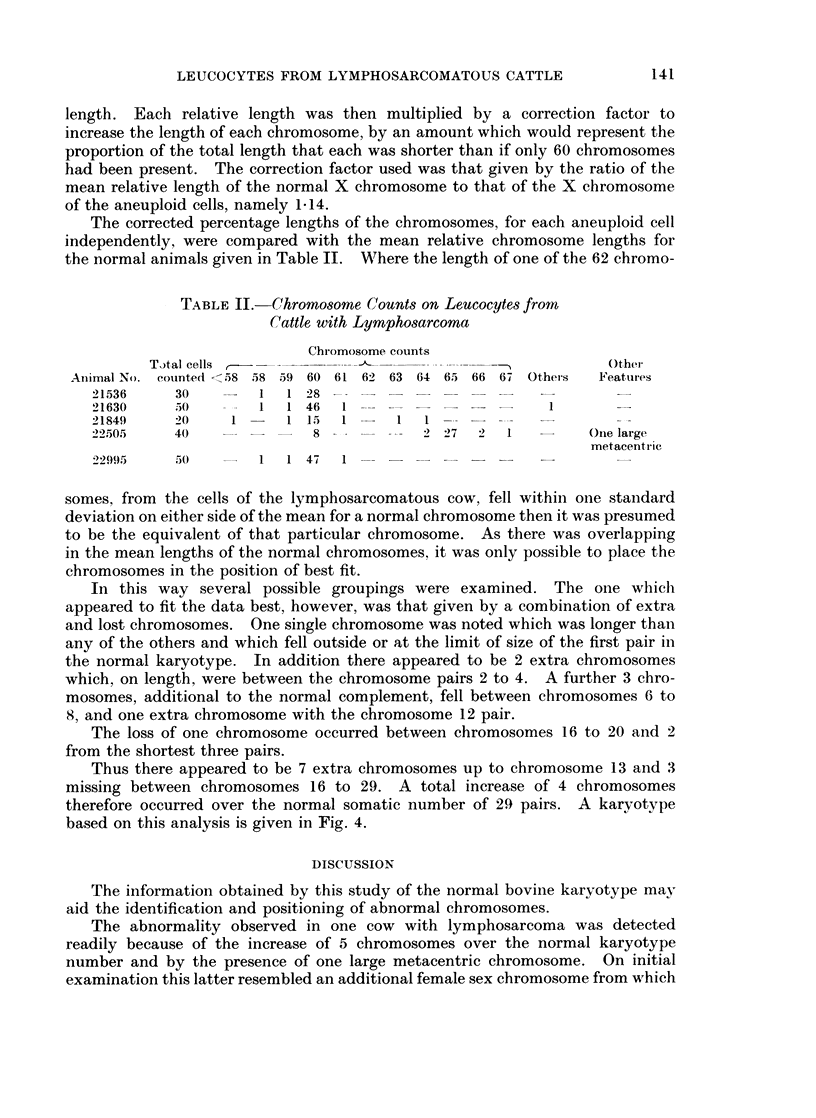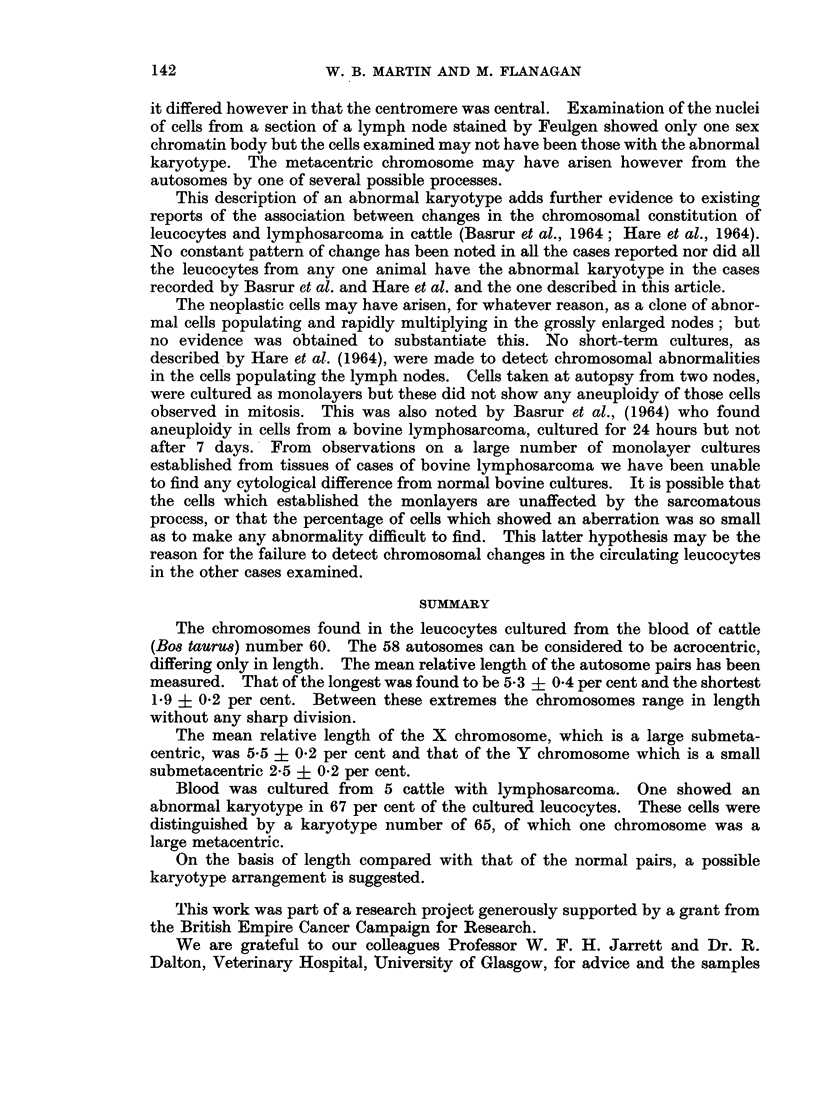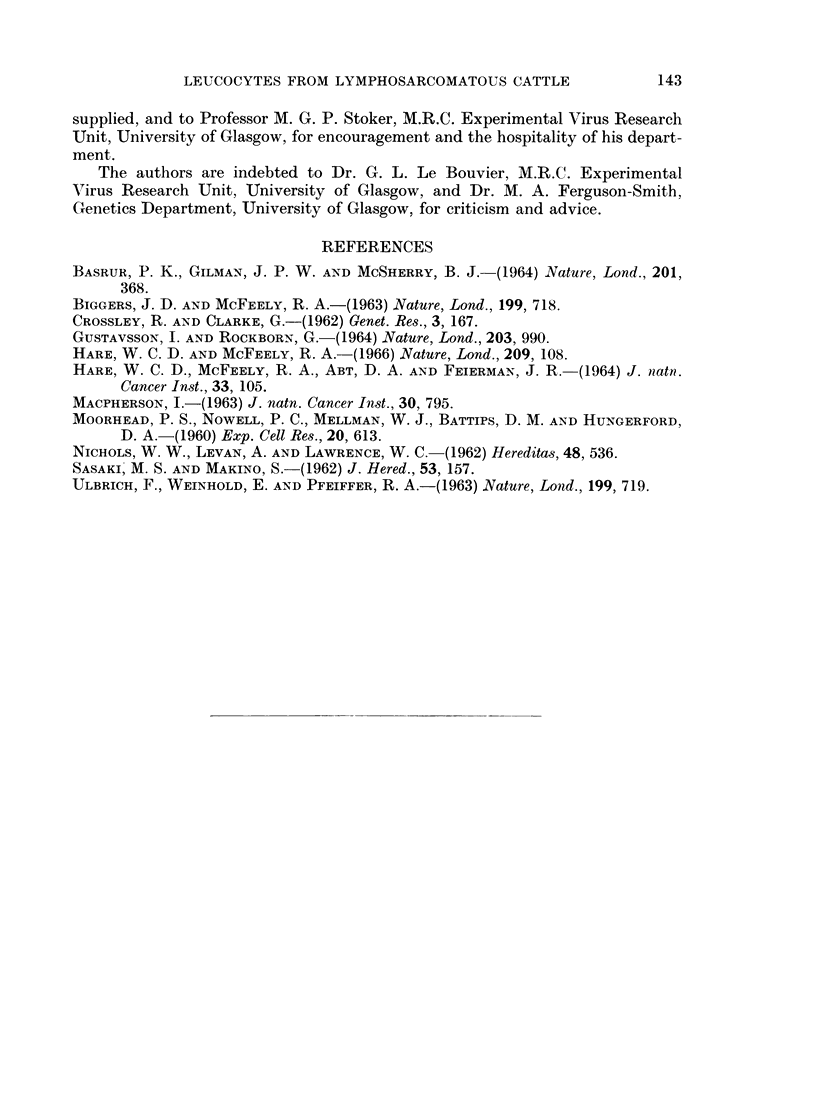# Karyotype analysis of leucocytes from normal and lymphosarcomatous cattle (Bos taurus).

**DOI:** 10.1038/bjc.1967.12

**Published:** 1967-03

**Authors:** W. B. Martin, M. Flanagan

## Abstract

**Images:**


### KARYOTYPE ANALYSIS OF LEUCOCYTES FROM NORMAL AND LYMPHOSARCOMATOUS CATTLE (*BOS TAURUS*)

W. B. MARTIN* AND M. FLANAGAN*

*From the Veterinary Hospital, University of Glasgow.*

Received for publication September 7, 1966

THE technique of peripheral blood culture using phytohaemagglutinin (Moorhead *et al.*, 1960) was described by Crossley and Clarke (1962) for the examination of bovine chromosomes. Since then several workers have recorded their variation of the technique as applied to cattle blood and have described the bovine karyotype (Nichols, Levan and Lawrence, 1962; Biggers and McFeely, 1963; Ulbrich, Weinhold and Pfeiffer, 1963). The normal somatic chromosome number of cattle (*Bos taurus*) has been reported as 60 (Crossley and Clarke, 1962; Sasaki and Makino, 1962; Ulbricht *et al.*, 1963).

As part of an investigation into lymphosarcoma of cattle in Scotland, the chromosomal constitution of circulating leucocytes from normal and affected cattle was studied. While this investigation was being conducted reports of unusual karyotypes found in the cells of cattle affected with lymphosarcoma were published from Canada (Basrur, Gilman and McSherry, 1964), America (Hare *et al.*, 1964), and Sweden (Gustavsson and Rockborn, 1964).

Basrur *et al.* (1964) reported a chromosomal abnormality in the cells from a lymph node of a cow with lymphosarcoma. Of the cells examined in a culture grown for 24 hours, 61 per cent carried one extra chromosome which resembled the X chromosome in being a large submetacentric.

Hare *et al.* (1964) recorded six cases of lymphosarcoma in which a varying proportion of cells cultured from lymph nodes showed changes in the chromosomal constitution. Two other cows exhibited aneuploidy in the leucocytes cultured from peripheral blood.

In three cows with lymphatic leukaemia studied by Gustavsson and Rockborn (1964), all the peripheral leucocytes examined showed 59 chromosomes. As all the cells showed an identical change, which was present also in the cells cultured from bone marrow and kidney of the foetus from one affected cow, Hare and McFeely (1966) have warned that the unusual chromosomal complement might have been inherited.

In our initial examinations of the chromosomes in cells obtained from peripheral blood cultures we were unable to find any obvious aberration. We attempted therefore to classify normal chromosomes by length so as to detect any fine differences. In this article details are given of the normal bovine chromosomes and of aneuploidy in one cow with lymphosarcoma.

* Present address: Department of Pathology, Faculty of Veterinary Science, University College, Nairobi, Kenya.

### MATERIALS AND METHODS

Individual sporadic cases of bovine lymphosarcoma occur in Scotland. Some of those presented at the University of Glasgow Veterinary Hospital had a sample of peripheral blood cultured for chromosome analysis. These animals, with few exceptions were not leukaemic. None came from multiple-case herds.

Samples of blood were taken into heparinised one ounce Universal bottles and centrifuged for 15 minutes at about 1000 **g**. After removing the plasma, the buffy coat was sucked off with a Pasteur pipette. It had been found that only exceptionally did phytohaemagglutinin assist separation and therefore separation of the white cells by centrifugation was adopted. Into 10 ml. of a modified Eagle's medium containing 20 per cent autologous plasma in a screw-capped Universal container were placed at least $10^6$ white cells. Several bottles of cells were prepared in this way, to each was added 0·2 ml. each of phytohaemagglutinin P & M (DIFCO LABS.) and the cultures incubated at 37° C. Generally at the third and fifth days of incubation 0·25 ml. of a 0·04 per cent solution of colcemid (CIBA LABS. LTD.) was added, and after about 2·5 to 5 hours the cells were centrifuged out, and resuspended in 1 per cent sodium citrate at 37° C. for 20 minutes. The cells were then fixed and stained by the method described by Macpherson (1963).

Only those slides showing a sufficient number of intact cells with well spaced chromosomes were considered suitable for analysis. The chromosomes, in such cells, were counted at least three times to determine the exact number.

To calculate the relative lengths of the chromosomes, cells with well delineated straight chromosomes were photographed. Suitable negatives were projected on paper and the outline of the chromosomes traced, noting as exactly as possible the extremities of each. The length of each arm was measured along the mid-line, and the mean length calculated for each two chromosomes which most closely matched. The relative length was the percentage each mean represented of the total female haploid length.

### RESULTS

We were able to confirm that the normal somatic chromosome number of *Bos taurus* was 60. This figure was based on counts made of the chromosomes in leucocytes from 6 normal cattle, 3 of each sex. The majority of cells had 60 chromosomes (Table I). A few cells in 3 animals gave counts slightly greater or

TABLE I.—*Chromosome Counts on Leucocytes from Normal Cattle*

| Animal No. | Total cells counted | Chromosome counts 58 | 59 | 60 | 61 | Others |
|---|---|---|---|---|---|---|
| 21699 | 18 | — | 3 | 14 | 1 | — |
| 21744 | 25 | 1 | — | 24 | — | — |
| 22370 | 25 | — | — | 25 | — | — |
| 22873 | 25 | — | — | 25 | — | — |
| N | 20 | 2 | 1 | 13 | 3 | 1 |
| No. 3 | 50 | — | — | 50 | — | — |

smaller than the diploid number. No reason for this spread was obvious and no significance was attached to it. The autosomal chromosomes of cattle differ from each other only in length, whereas the two sex chromosomes are readily identifiable

(Fig. 1A and 1B). The X chromosome is a large submetacentric and the Y a small submetacentric chromosome.

The majority of the 58 autosomes have centromeres which appeared to be terminal, though occasional cells in some preparations showed chromosomes with distinct short arms and subterminal centromeres. These were not sufficiently consistent within or between preparations to allow regular unequivocal identification of individual chromosomes. It may well be that the centromeres are not situated at the extreme tip and that very short arms are present in all chromosomes but are so small as to be frequently hidden as suggested by Crossley and Clarke (1962). Like them, we believe that the autosomes should be considered to be acrocentric.

As no other suitable feature could be found to identify pairs of autosomes, these had to be assorted on the basis of size alone. The mean relative length for each of the 29 pairs of autosomes has been calculated from a total of 10 mitotic figures seen in peripheral leucocyte cultures from 5 different normal individuals, of which 2 were males. The means and deviations for each pair of chromosomes are given in Fig. 2. It can be seen that the longest autosomal chromosome is

FIG. 2.—The Normal Bovine Idiogram : giving the mean relative length of each pair of autosomes and the sex chromosomes.

$5 \cdot 3 \pm 0 \cdot 4$ per cent and the shortest $1 \cdot 9 \pm 0 \cdot 2$ per cent of the total female haploid length. Between these two extremes the other autosomes range in length without a sufficiently clear division to allow separation into groups.

The mean relative length of the X chromosome was found to be $5 \cdot 5 \pm 0 \cdot 2$ per cent, which made it comparable in length to the longest autosomal pair. In contrast the Y chromsome was very short, representing only about $2 \cdot 5 \pm 0 \cdot 2$ per cent and, as pointed out by Sasaki and Makino (1962), approximately the length of the twenty-third to twenty-fifth pairs. The arm ratio of the X chromosome averaged 1·7 and that of the Y 1·1.

Peripheral leucocyte cultures were prepared from cattle affected with lymphosarcoma, and the chromosomes compared with those from normal cattle. None of the sarcomatous animals was leukaemic at the time of examination. The number and features of the chromosomes showed no characteristic or regular deviation from the normal except in one cow (22505), also not leukaemic, in which some of the cells were aneuploid (Table II).

About 67 per cent of the cultured cells from cow 22505 had a chromosome number of 65 and only 20 per cent a chromosome number of 60. The variation around 65 was no greater than occurred around 60 in normal or other lymphosarcoma animals. Another important feature was the presence of a single large metacentric chromosome about equal in length to the X chromosomes but distinguishable from them by the position of the centromere. This metacentric chromosome was only present in the cells showing the deviation in number from 60. Those cells with 60 chromosomes were apparently normal in appearance. The chromosomes in two representative cells, illustrated in Fig. 3A and 3B, were examined in detail. The mean length of the metacentric chromosomes was only 0·15 units greater than the mean length of the X chromosomes from the same two cells. The mean arm ratio of these X chromosomes, however, was 1·5 in contrast with that of the metacentric chromosomes which was 1·06.

In the cells showing 65 chromosomes, 3 chromosomes could thus be readily identified, namely the 2 sex chromosomes and the large metacentric. The remaining 62 autosomes apparently all had terminal centromeres and were distinguishable only by size. In order to allow further analysis of these chromosomes it was presumed that 4 chromosomes only were extra. Though obviously a combination of extra and lost chromosomes might result in an excess of 4 chromosomes, it seemed reasonable to consider the simplest hypothesis that there were present only extra chromosomes without any missing.

The chromosomes present in the two cells typical of those showing 65 chromosomes were measured. The relative length of each of the 62 acrocentric autosomes was calculated without the addition of the metacentric chromosome to the total

---

EXPLANATION OF PLATES

FIG. 1A.—*Bos taurus* : chromosomes of a female leucocyte in mitosis. (Sex chromosomes marked X).

FIG. 1B.—*Bos taurus* : chromosomes of a male leucocyte in mitosis. (Sex chromosomes marked X and Y).

FIG. 3A and 3B.—Chromosomes of two leucocytes from a cow (22505) with lymphosarcoma (Female sex chromosomes marked X, and the single metacentric chromosome marked M).

FIG. 4.—A possible arrangement of the chromosomes of the leucocytes from a cow with lymphosarcoma.

X
X
1A
X
Y
1B

3A

3B

Martin and Flanagan.

1

2

3

4

5

6

7

8

9

10

11

12

13

14

15

16

17

18

19

20

21

22

23

24

25

26

27

28

29

X

X

4A

4

length. Each relative length was then multiplied by a correction factor to increase the length of each chromosome, by an amount which would represent the proportion of the total length that each was shorter than if only 60 chromosomes had been present. The correction factor used was that given by the ratio of the mean relative length of the normal X chromosome to that of the X chromosome of the aneuploid cells, namely 1·14.

The corrected percentage lengths of the chromosomes, for each aneuploid cell independently, were compared with the mean relative chromosome lengths for the normal animals given in Table II. Where the length of one of the 62 chromo-

TABLE II.—*Chromosome Counts on Leucocytes from Cattle with Lymphosarcoma*

| Animal No. | Total cells counted | Chromosome counts <58 | 58 | 59 | 60 | 61 | 62 | 63 | 64 | 65 | 66 | 67 | Others | Other Features |
|---|---|---|---|---|---|---|---|---|---|---|---|---|---|---|
| 21536 | 30 | — | 1 | 1 | 28 | — | — | — | — | — | — | — | — | — |
| 21630 | 50 | — | 1 | 1 | 46 | 1 | — | — | — | — | — | — | 1 | — |
| 21849 | 20 | 1 | — | 1 | 15 | 1 | — | 1 | 1 | — | — | — | — | — |
| 22505 | 40 | — | — | — | 8 | — | — | — | 2 | 27 | 2 | 1 | — | One large metacentric |
| 22995 | 50 | — | 1 | 1 | 47 | 1 | — | — | — | — | — | — | — | — |

somes, from the cells of the lymphosarcomatous cow, fell within one standard deviation on either side of the mean for a normal chromosome then it was presumed to be the equivalent of that particular chromosome. As there was overlapping in the mean lengths of the normal chromosomes, it was only possible to place the chromosomes in the position of best fit.

In this way several possible groupings were examined. The one which appeared to fit the data best, however, was that given by a combination of extra and lost chromosomes. One single chromosome was noted which was longer than any of the others and which fell outside or at the limit of size of the first pair in the normal karyotype. In addition there appeared to be 2 extra chromosomes which, on length, were between the chromosome pairs 2 to 4. A further 3 chromosomes, additional to the normal complement, fell between chromosomes 6 to 8, and one extra chromosome with the chromosome 12 pair.

The loss of one chromosome occurred between chromosomes 16 to 20 and 2 from the shortest three pairs.

Thus there appeared to be 7 extra chromosomes up to chromosome 13 and 3 missing between chromosomes 16 to 29. A total increase of 4 chromosomes therefore occurred over the normal somatic number of 29 pairs. A karyotype based on this analysis is given in Fig. 4.

### DISCUSSION

The information obtained by this study of the normal bovine karyotype may aid the identification and positioning of abnormal chromosomes.

The abnormality observed in one cow with lymphosarcoma was detected readily because of the increase of 5 chromosomes over the normal karyotype number and by the presence of one large metacentric chromosome. On initial examination this latter resembled an additional female sex chromosome from which it differed however in that the centromere was central. Examination of the nuclei of cells from a section of a lymph node stained by Feulgen showed only one sex chromatin body but the cells examined may not have been those with the abnormal karyotype. The metacentric chromosome may have arisen however from the autosomes by one of several possible processes.

This description of an abnormal karyotype adds further evidence to existing reports of the association between changes in the chromosomal constitution of leucocytes and lymphosarcoma in cattle (Basrur *et al.*, 1964 ; Hare *et al.*, 1964). No constant pattern of change has been noted in all the cases reported nor did all the leucocytes from any one animal have the abnormal karyotype in the cases recorded by Basrur *et al.* and Hare *et al.* and the one described in this article.

The neoplastic cells may have arisen, for whatever reason, as a clone of abnormal cells populating and rapidly multiplying in the grossly enlarged nodes ; but no evidence was obtained to substantiate this. No short-term cultures, as described by Hare *et al.* (1964), were made to detect chromosomal abnormalities in the cells populating the lymph nodes. Cells taken at autopsy from two nodes, were cultured as monolayers but these did not show any aneuploidy of those cells observed in mitosis. This was also noted by Basrur *et al.*, (1964) who found aneuploidy in cells from a bovine lymphosarcoma, cultured for 24 hours but not after 7 days. From observations on a large number of monolayer cultures established from tissues of cases of bovine lymphosarcoma we have been unable to find any cytological difference from normal bovine cultures. It is possible that the cells which established the monlayers are unaffected by the sarcomatous process, or that the percentage of cells which showed an aberration was so small as to make any abnormality difficult to find. This latter hypothesis may be the reason for the failure to detect chromosomal changes in the circulating leucocytes in the other cases examined.

### SUMMARY

The chromosomes found in the leucocytes cultured from the blood of cattle (*Bos taurus*) number 60. The 58 autosomes can be considered to be acrocentric, differing only in length. The mean relative length of the autosome pairs has been measured. That of the longest was found to be $5 \cdot 3 \pm 0 \cdot 4$ per cent and the shortest $1 \cdot 9 \pm 0 \cdot 2$ per cent. Between these extremes the chromosomes range in length without any sharp division.

The mean relative length of the X chromosome, which is a large submetacentric, was $5 \cdot 5 \pm 0 \cdot 2$ per cent and that of the Y chromosome which is a small submetacentric $2 \cdot 5 \pm 0 \cdot 2$ per cent.

Blood was cultured from 5 cattle with lymphosarcoma. One showed an abnormal karyotype in 67 per cent of the cultured leucocytes. These cells were distinguished by a karyotype number of 65, of which one chromosome was a large metacentric.

On the basis of length compared with that of the normal pairs, a possible karyotype arrangement is suggested.

This work was part of a research project generously supported by a grant from the British Empire Cancer Campaign for Research.

We are grateful to our colleagues Professor W. F. H. Jarrett and Dr. R. Dalton, Veterinary Hospital, University of Glasgow, for advice and the samples supplied, and to Professor M. G. P. Stoker, M.R.C. Experimental Virus Research Unit, University of Glasgow, for encouragement and the hospitality of his department.

The authors are indebted to Dr. G. L. Le Bouvier, M.R.C. Experimental Virus Research Unit, University of Glasgow, and Dr. M. A. Ferguson-Smith, Genetics Department, University of Glasgow, for criticism and advice.

### REFERENCES

Basrur, P. K., Gilman, J. P. W. and McSherry, B. J.—(1964) *Nature, Lond.*, **201**, 368.

Biggers, J. D. and McFeely, R. A.—(1963) *Nature, Lond.*, **199**, 718.

Crossley, R. and Clarke, G.—(1962) *Genet. Res.*, **3**, 167.

Gustavsson, I. and Rockborn, G.—(1964) *Nature, Lond.*, **203**, 990.

Hare, W. C. D. and McFeely, R. A.—(1966) *Nature, Lond.*, **209**, 108.

Hare, W. C. D., McFeely, R. A., Abt, D. A. and Feierman, J. R.—(1964) *J. natn. Cancer Inst.*, **33**, 105.

Macpherson, I.—(1963) *J. natn. Cancer Inst.*, **30**, 795.

Moorhead, P. S., Nowell, P. C., Mellman, W. J., Battips, D. M. and Hungerford, D. A.—(1960) *Exp. Cell Res.*, **20**, 613.

Nichols, W. W., Levan, A. and Lawrence, W. C.—(1962) *Hereditas*, **48**, 536.

Sasaki, M. S. and Makino, S.—(1962) *J. Hered.*, **53**, 157.

Ulbrich, F., Weinhold, E. and Pfeiffer, R. A.—(1963) *Nature, Lond.*, **199**, 719.